# Dragonfly-Wing-Inspired Bluff-Body Piezoelectric Harvester for Efficient Low-Wind-Speed Energy Harvesting

**DOI:** 10.3390/mi17030380

**Published:** 2026-03-20

**Authors:** Zhiyong Zhou, Xinyu Shang, Yebao Xia, Pei Zhu

**Affiliations:** 1 School of Civil and Architectural Engineering, Henan University, Kaifeng 475004, China; 2 AVIC Nanjing Engineering Institute of Aircraft Systems, Nanjing 211106, China

**Keywords:** dragonfly-inspired bluff body, piezoelectric wind energy harvester, low wind speed, vortex-induced vibration, galloping, bio-inspired design, CFD simulation

## Abstract

Inspired by the wing-opening morphology of dragonflies, a series of bio-inspired dragonfly-shaped bluff bodies are designed and investigated, and further integrated into a piezoelectric wind energy harvester. The energy-harvesting performance and aerodynamic responses of bluff-body configurations with different wing-opening angles (0°, 15°, 30°, 45°, and 60°) are comparatively analyzed through a combination of numerical simulations and wind tunnel experiments. Experimental results demonstrate pronounced differences among the configurations in the low wind speed regime. Specifically, the prototype with α = 0° achieves relatively higher output under very low wind speeds, whereas the α = 15° configuration exhibits the best overall performance across the entire tested wind speed range. Taking the α = 15° case as an example, the cut-in wind speed is reduced to 1.7 m/s, while the maximum RMS voltage and output power are increased by 20.16% and 44.39% compared with the cuboid bluff body, and by 50.95% and 127.84% compared with the cylinder bluff body, respectively. Further CFD results reveal that, at specific wing-opening angles, the dragonfly-shaped bluff body undergoes a coupled vortex-induced vibration (VIV) and galloping response, enabling certain configurations to sustain stable oscillations with large amplitudes over a relatively wide wind speed range. Within the investigated parameter range, an appropriate selection of the wing-opening angle effectively balances the cut-in capability and output stability under low wind speed conditions. These findings provide useful design guidelines for flow-induced vibration-based wind energy harvesters operating in low wind speed environments.

## 1. Introduction

In recent years, rapid advances in digital technologies have accelerated the transition of the Internet of Things (IoT) from conceptual exploration to practical engineering applications. Enabled by microelectronic devices and wireless sensor networks, IoT systems are capable of continuously acquiring environmental and structural information for remote monitoring and intelligent management [1,2,3]. However, when deployed at large scale in remote or complex environments, sensor nodes are commonly constrained by battery-based power supply, which leads to high maintenance costs and limited long-term operational stability, thereby restricting system reliability and scalability [4,5,6,7]. Among available distributed energy solutions, wind energy is particularly attractive owing to its flexible accessibility and environmental adaptability. Given that ambient wind in most real-world scenarios is typically characterized by low speeds and pronounced fluctuations, stable energy harvesting under low wind speed conditions has become an important research topic for sustainable IoT power supply [8,9,10,11,12,13,14].

Compared with conventional small-scale wind turbines, flow-induced vibration (FIV)-based wind energy harvesters can more readily establish effective energy input under low wind speed conditions and have therefore received increasing attention [15,16]. Rather than relying on continuous aerodynamic torque, FIV-based devices utilize unsteady flow–structure interactions to convert wind energy into structural vibrations, which are subsequently transformed into electrical energy through electromechanical coupling, forming a continuous energy conversion process [17,18,19]. In existing studies, FIV mainly appears in the form of VIV [20,21,22], galloping [23,24,25], and flutter [26,27,28]. VIV-based harvesters benefit from the lock-in effect and can achieve relatively high output at low wind speeds; however, their effective operating bandwidth is typically narrow, and performance deteriorates rapidly once the wind speed exceeds the lock-in region. By contrast, galloping-based harvesters arise from aeroelastic instability and are capable of inducing large-amplitude oscillations at low wind speeds while maintaining enhanced responses as wind speed increases, making them more suitable for operation over a broader wind speed range [29,30,31].

With respect to galloping-based energy harvesting, bluff-body geometry is widely regarded as a decisive factor governing aerodynamic excitation and energy conversion efficiency. Xia et al. [32] enhanced aerodynamic instability by introducing symmetric flexible splitter plates on both sides of a cuboid bluff body, demonstrating that both cut-in performance and electrical output can be significantly improved at appropriate installation angles. In studies closer to engineering practice, Xue et al. [33] integrated a galloping energy harvester into a railway wind barrier structure and exploited locally accelerated airflow within internal channels to enhance aerodynamic excitation, resulting in a pronounced increase in output power under realistic operating conditions. To address the directional sensitivity of galloping-based harvesters, Xia et al. [34] proposed a multimodal configuration combining a tri-section beam with a splitter-plate-equipped cuboid bluff body, enabling effective vibration responses under varying wind directions, with higher-order bending modes exhibiting improved energy conversion efficiency. In addition, Zheng et al. [35] modified the windward geometry using a W-shaped bluff body, which effectively reduced the cut-in threshold at low wind speeds and achieved stable power output over a moderate wind speed range. From the perspective of surface-detail regulation, Xing et al. [36] systematically investigated leeward-side protrusions and showed that appropriately shaped protrusions, particularly elliptical ones, can significantly enhance aerodynamic excitation and output performance. Collectively, these studies highlight the critical role of bluff-body design in galloping-based energy harvesting, while challenges remain in simultaneously balancing cut-in capability, output stability, and operational applicability under low wind speed conditions.

At the mechanism level, several efforts have been devoted to overcoming the inherent limitations of conventional VIV-based harvesters. Dong et al. [37] proposed an internal-resonance energy harvester based on the coupling between VIV and flutter, in which multimodal interactions enabled pronounced energy amplification near the cut-in point. By incorporating an aero-electromechanical coupled model, the associated energy transfer pathways under internal resonance conditions were systematically examined, offering insights into extending the performance limits of VIV-based systems. Ma et al. [38] introduced a tristable VIV energy harvester and investigated the influence of base excitation on system response characteristics. From a geometric optimization perspective, Wang et al. [39] actively controlled flow separation by cutting the leading edge of a cylinder, thereby enhancing vibration amplitude and broadening the effective VIV operating range. Using a similar concept, Wang et al. further modified wake vortex evolution by etching concave metasurface structures on the cylinder surface, achieving either enhancement or suppression of VIV and clarifying the roles of load resistance and electromechanical coupling parameters through theoretical analysis [40].

As research has progressed, it has become increasingly evident that energy harvesters relying exclusively on either VIV or galloping suffer from intrinsically limited operating ranges. To address this issue, several studies have examined both mechanisms within a unified framework in order to exploit their complementary characteristics across different wind speed regimes. Zhang et al. [41] introduced windward and leeward attachments on a cylinder bluff body to induce a flow-driven transition from VIV to galloping, with a dual-windward configuration exhibiting higher electrical output at moderate wind speeds. Similarly, Wang et al. [42] employed asymmetric splitter plates to alter vortex shedding and actively trigger galloping responses, thereby significantly expanding the effective operating bandwidth. With a stronger emphasis on mechanism-oriented analysis, Andrianne et al. [43] incorporated both VIV and galloping characteristics of a square-beam structure into a unified framework and systematically examined the dynamic responses under different aeroelastic instability modes. Meanwhile, the influence of bluff-body cross-sectional geometry on the VIV–galloping transition process has also been explored. Wang et al. [44] investigated the transverse and pivoted vibration characteristics of a trapezoidal prism under different Reynolds numbers, revealing large vibration amplitudes during the VIV-to-galloping transition and improved energy harvesting efficiency at low Reynolds numbers. Building on this concept, Sun et al. [45] proposed a bulbous-section cylinder to further enhance VIV–galloping synergy, achieving notable performance improvement under low wind speed conditions.

With growing recognition of VIV–galloping synergistic mechanisms, recent research has shifted from purely dynamical regulation toward structural-level optimization of aerodynamic excitation, among which bio-inspired design has emerged as a particularly promising pathway. By incorporating representative geometric features evolved in biological organisms operating under low-Reynolds-number environments, bio-inspired structures have demonstrated reduced cut-in thresholds and enhanced output stability without the need for complex control strategies. Along this line, Zhu et al. [46] proposed a bio-inspired lift–drag hybrid turbine capable of initiating operation at low wind speeds and achieving high energy conversion efficiency at moderate wind speeds. Li et al. [47] introduced fractal geometry into bluff-body design, effectively reducing the galloping critical wind speed and enhancing output performance through increased wake complexity. The swallowtail-inspired V-shaped attachments proposed by Zhou et al. [48] and the corrugated splitter plates inspired by dragonfly wing veins designed by Guo et al. [49] further demonstrated superior low-wind-speed performance, which was primarily attributed to stronger and more stable vortex shedding. In addition, the Koch snowflake-inspired bluff body proposed by Li et al. [50] extended the VIV lock-in range and promoted the transition from VIV to galloping, leading to further improvement in overall energy harvesting performance. Beyond fixed geometries, adaptive and multimodal bio-inspired designs have also attracted attention. Liu et al. [51] proposed a dual-airfoil bluff body with adjustable attack angles, exhibiting multiple response modes and achieving higher energy output under appropriate inflow conditions. Zhu et al. [52] developed a bio-inspired bladed hybrid generator by mimicking the coordinated motion of dolphin dorsal fins and tails, enabling stable power generation at low wind speeds and successfully powering practical sensor nodes. Moreover, the bio-inspired spider structure proposed by Zhou et al. [53] demonstrated the potential of bio-inspired architectures for broadband vibration energy harvesting.

Dragonfly wings are slender natural structures characterized by elongated planforms, whose geometries are representative of flying bodies operating under low Reynolds number conditions. Such wings typically feature relatively sharp leading edges, gradually tapered trailing edges, and high aspect ratios, making them suitable references for structural biomimicry and engineering abstraction. Accordingly, rather than directly reproducing realistic wing morphology, the present study extracts representative geometric features from the dragonfly wing-spreading configuration and incorporates them into the design of a bluff body. Based on this concept, a bio-inspired dragonfly-shaped piezoelectric energy harvester (BDPEH) is developed. By introducing symmetric wing-like plates into the bluff-body cross section and defining the wing-opening angle as a tunable parameter, a series of prototypes with identical overall dimensions are constructed to systematically investigate aerodynamic responses, vibration behavior, and energy harvesting performance under low wind speed conditions.

In this study, a dragonfly-wing-inspired bluff body with adjustable wing-opening angle was proposed for piezoelectric wind energy harvesting at low wind speeds. Compared with previous studies that mainly focused on fixed bio-inspired geometries, the present work treats the wing-opening angle as a key structural parameter and examines its effects on aerodynamic excitation, vibration response, and energy harvesting performance. The results provide a useful reference for the design of low-wind-speed flow-induced vibration energy harvesters.

Section 2 introduces the BDPEH design and experimental setup. Section 3 reports experimental results under different wing-opening angles with comparisons to cuboid and cylinder bluff bodies. Section 4 presents CFD analysis of the flow field and vortex shedding, followed by conclusions in Section 5.

## 2. Design and Mechanism of the Bio-Inspired Bluff Body

**Figure 1:**
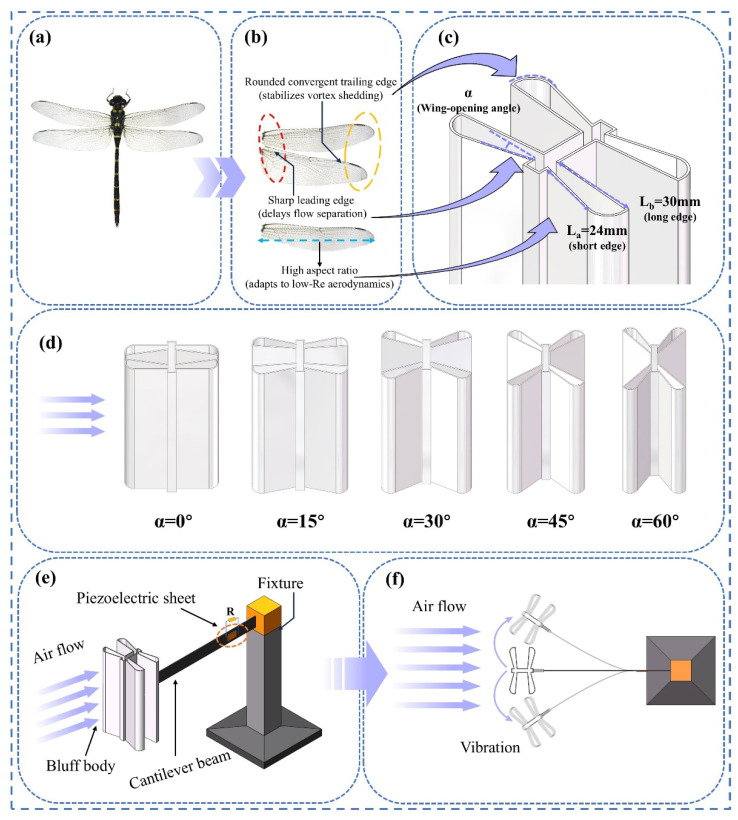
Design concept and operating mechanism of the dragonfly-wing-inspired piezoelectric energy harvester (BDPEH): (**a**) bio-inspired prototype of the dragonfly wing; (**b**) extracted aerodynamic features including sharp leading edges, converging trailing edges, and high aspect ratio; (**c**) parametric geometry and definition of the spreading angle α; (**d**) cross-sectional configurations with different spreading angles (α = 0–60°); (**e**) photograph of the BDPEH prototype; (**f**) schematic illustration of the vibration-induced energy harvesting mechanism.

Figure 1 illustrates the overall design concept of the bio-inspired dragonfly-shaped piezoelectric energy harvester (BDPEH) and the source of inspiration for its geometric configuration. As shown in Figure 1a, dragonfly wings are selected as the bio-inspired prototype, whose morphological characteristics are mainly reflected by a slender wing-spreading profile, a relatively sharp leading edge, and a gradually tapered trailing edge. Such geometric features are commonly observed in flying bodies operating under low Reynolds number conditions and are therefore adopted as a reference for engineering abstraction and structural reconstruction in this study. In the specific bluff-body design, the realistic wing morphology is not directly reproduced. Instead, its geometric features are simplified and transformed into a parameterized bluff-body cross-sectional configuration. The windward leading edge consists of two intersecting edges with different lengths (L_a_ = 24 mm and L_b_ = 30 mm), forming an included angle of 15°, and is smoothly connected by a circular arc with a radius of 6.1 mm to ensure a continuous leading-edge profile. The central region is simplified as a rectangular structure with dimensions of 5 mm × 30 mm, serving as the primary flow-interaction component and forming a stable geometric connection with the leading-edge wing plates. A transition distance of 3.5 mm is reserved from the junction between the short leading-edge side and the central structure to the top edge, ensuring geometric continuity and structural stability of the overall configuration. While maintaining the aforementioned basic configuration unchanged, the wing-opening angle α is introduced at the junction between the leading-edge wing plates and the central structure as the primary adjustable parameter. By varying α, a series of bluff-body geometries can be generated without altering the wing dimensions, providing a unified parameterized design framework for systematically comparing aerodynamic responses, vibration characteristics, and energy harvesting performance under different α values, and for identifying the optimal dragonfly-inspired bluff-body configuration.

Figure 1b illustrates the geometric configurations of the BDPEH under different wing-opening angles (α = 0°, 15°, 30°, 45°, and 60°). The bluff body consists of two wings symmetrically arranged about the central body, where the wing-opening angle α is defined as the angle between the short edge L_a_ of each wing and the central rectangular section. By varying α while keeping the main dimensions unchanged, a series of bluff bodies with different geometric configurations are generated, reflecting the influence of wing-spreading morphology on the flow-interaction configuration. Figure 1c presents a schematic of the BDPEH bluff-body design, in which the key geometric parameters L_a_, L_b_, and the definition of the wing-opening angle α are indicated, providing an intuitive illustration of the engineering transformation from dragonfly wing-spreading geometry to a bluff-body structure. Figure 1d–f show the experimental setup and working mechanism of the BDPEH. Figure 1d displays the cross-sectional configurations of the BDPEH under different wing-opening angles α, while Figure 1e presents the structural components of the BDPEH prototype, including the cantilever beam, piezoelectric patches, and the dragonfly-shaped bluff body. The bluff body is mounted at the free end of the cantilever beam, and the fixed end is clamped to the supporting structure. Figure 1f schematically illustrates the working mechanism of the BDPEH; when the incoming flow acts on the bluff body, alternating unsteady aerodynamic forces are generated, driving transverse vibrations of the cantilever beam, which induce periodic deformation of the piezoelectric patches and consequently generate electrical output.

In this study, while keeping the main wing dimensions unchanged, five wing-opening angles (α = 0°, 15°, 30°, 45°, and 60°) are selected to represent different degrees of wing spreading. These configurations constitute a set of representative geometric samples for subsequent comparative analyses of aerodynamic responses and energy harvesting performance under different wing-opening conditions.

To describe the overall vibration behavior and voltage output of the BDPEH under different wind speed conditions, the aerodynamic, structural, and electrical responses of the system are treated using an equivalent modeling approach. Subsequent experimental results indicate that the system vibration is primarily dominated by the first bending mode of the cantilever beam, and the influence of different bluff-body configurations on the overall response can be effectively interpreted through this dominant mode. Based on this observation, a lumped-parameter approach is adopted, in which the BDPEH is equivalently modeled as a single-degree-of-freedom (SDOF) electromechanically coupled system to capture its primary dynamic characteristics. In this equivalent model, the cantilever beam–bluff body assembly is simplified as a mass–spring–damper system to represent its transverse vibration behavior, while the electromechanical transduction of the piezoelectric patch is incorporated through an equivalent capacitance and an external load resistance, thereby establishing the coupling between structural vibration and voltage output.

Accordingly, the electromechanically coupled governing equations describing the relationship between the transverse displacement and the piezoelectric voltage of the BDPEH can be derived, which are expressed as follows [54,55]:

(1) $$m\ddot{y}\left(t\right)+c\dot{y}(t)+ky(t)+\theta V(t)=F_{y}(t)$$

(2) $$C_{p}\dot{V}\left(t\right)+\frac{V\left(t\right)}{R}=\theta \dot{y}\left(t\right)$$

where $y\left(t\right)$ is the transverse displacement of the bluff body; $V\left(t\right)$ the piezoelectric voltage; m,c,k denote the equivalent mass, damping, and stiffness coefficients, respectively; θ is the electromechanical coupling coefficient; $C_{p}$ the capacitance; and R the external load resistance. The aerodynamic force $F_{y}$ primarily includes the coupled contributions of galloping and vortex-induced excitations acting at the bluff body’s center of mass, and can be expressed as [56]:

(3) $$F_{y}=-\frac{1}{2}\rho U^{2}WH\left(C_{L}+C_{D}\tan\emptyset \right)\sec\emptyset$$

where ρ is the air density, U the inflow velocity, W and H the width and height of the bluff body, respectively; $C_{L}$ and $C_{D}$ are the lift and drag coefficients; and ∅ denotes the effective angle of attack.

The above aerodynamic force model is primarily employed to characterize the fundamental features of aerodynamic excitation associated with different bluff-body configurations. Within a unified modeling framework, Equations (1)–(3) describe the structural vibration, electromechanical coupling, and aerodynamic excitation of the BDPEH, thereby providing a theoretical basis for interpreting the experimental observations and CFD results presented in subsequent sections.

## 3. Experimental Methods and Comparative Study

### 3.1. Experimental Setup and Test Platform

**Figure 2:**
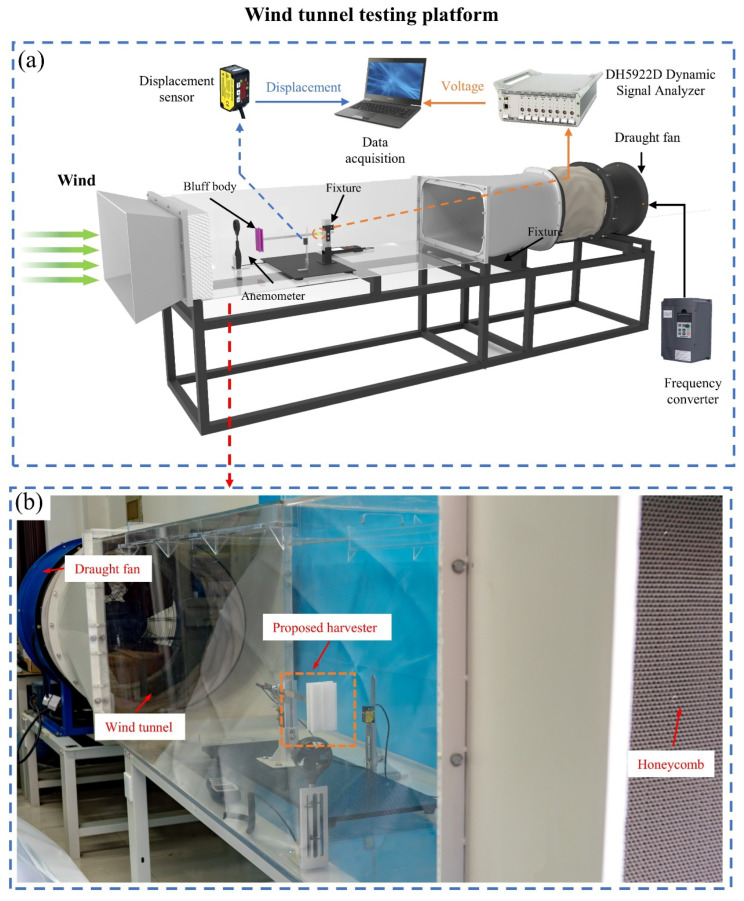
Wind tunnel experimental setup used for performance evaluation: (**a**) schematic diagram of the wind tunnel and data acquisition system; (**b**) photograph of the experimental platform.

To validate and evaluate the energy harvesting performance of the BDPEH, comparative experiments are conducted using the wind tunnel test platform shown in Figure 2. The experimental system mainly consists of a wind tunnel facility, energy harvester prototypes, a frequency control unit, and a data acquisition system (DH5922D, Donghua). The rotational speed of the fan inside the wind tunnel is regulated by a frequency inverter, enabling continuous control of the incoming wind speed. The prototype is installed in a rectangular test section, where a honeycomb flow straightener is placed upstream to improve flow uniformity, and an exhaust fan is arranged downstream to maintain stable inflow conditions.

During the experiments, the incoming wind speed is monitored in real time using a digital anemometer (AS8336, Smart Sensor Ltd. Smart Sensor, Holding Company Ltd., Dongguan, China) and is coordinated with the fan control system. Under airflow excitation, the bluff body drives transverse vibrations of the cantilever beam, inducing periodic deformation of the piezoelectric patch and generating electrical output. A laser displacement sensor (HG-C1100, Panasonic, Tokyo, Japan) is positioned near the piezoelectric patch to measure the transverse displacement response of the cantilever beam, while a high-precision dynamic signal analyzer simultaneously acquires the displacement signals and the output voltage of the piezoelectric patch.

**Figure 3:**
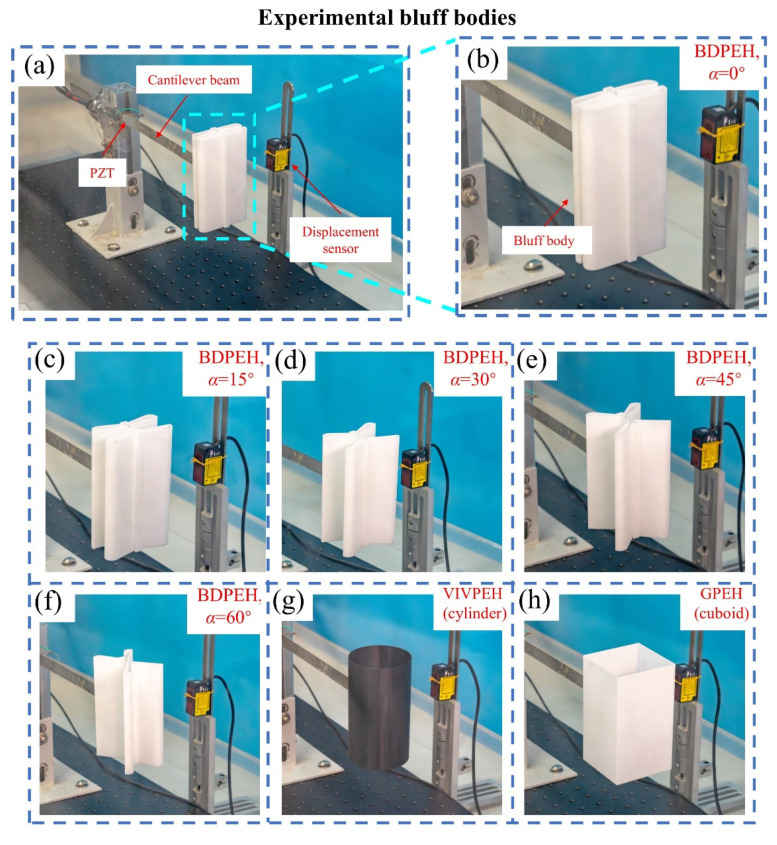
Bluff-body configurations investigated in the wind tunnel experiments: (**a**) installation of the cantilever beam, piezoelectric patch (PZT), and displacement sensor; (**b**) BDPEH (α = 0°); (**c**) BDPEH (α = 15°); (**d**) BDPEH (α = 30°); (**e**) BDPEH (α = 45°); (**f**) BDPEH (α = 60°); (**g**) circular cylinder (VIVPEH); (**h**) square prism (GPEH).

Three types of bluff-body configurations are considered for comparison to systematically evaluate the energy harvesting performance, including the bio-inspired dragonfly-shaped bluff body (BDPEH), the cuboid bluff body (GPEH), and the cylinder bluff body (VIVPEH). The geometric dimensions of the GPEH and VIVPEH are designed to be identical to those of the BDPEH with α = 15° to ensure fair comparison. Specifically, the cuboid cross section measures 66.77 mm × 120 mm, the cylinder has a diameter of 66.77 mm, and both bluff bodies have a height of 120 mm with a wall thickness of 0.8 mm. All bluff bodies are fabricated from PLA material using 3D printing and are uniformly mounted at the free end of a cantilever beam with dimensions of 205 mm × 12 mm × 1 mm. The configurations and installation of the bluff bodies are shown in Figure 3, including five BDPEHs with different wing-opening angles (α = 0°, 15°, 30°, 45°, and 60°), the VIVPEH, and the GPEH.

### 3.2. Performance Comparison and Results

In the dragonfly-shaped bluff body, the wing-opening angle α characterizes the degree of opening between the upper and lower wings relative to the central body and serves as a key geometric parameter distinguishing different configurations. As α varies, both the windward projected shape and the cross-sectional profile of the bluff body change accordingly, leading to distinct geometrical configurations. Based on these geometric differences, five wing-opening angles (α = 0°, 15°, 30°, 45°, and 60°) are selected to construct a set of dragonfly-shaped bluff-body prototypes with identical overall dimensions but different opening degrees. Comparative experiments are then conducted against the conventional cuboid bluff body (GPEH) and cylinder bluff body (VIVPEH) to evaluate the energy harvesting performance under different configurations.

**Figure 4:**
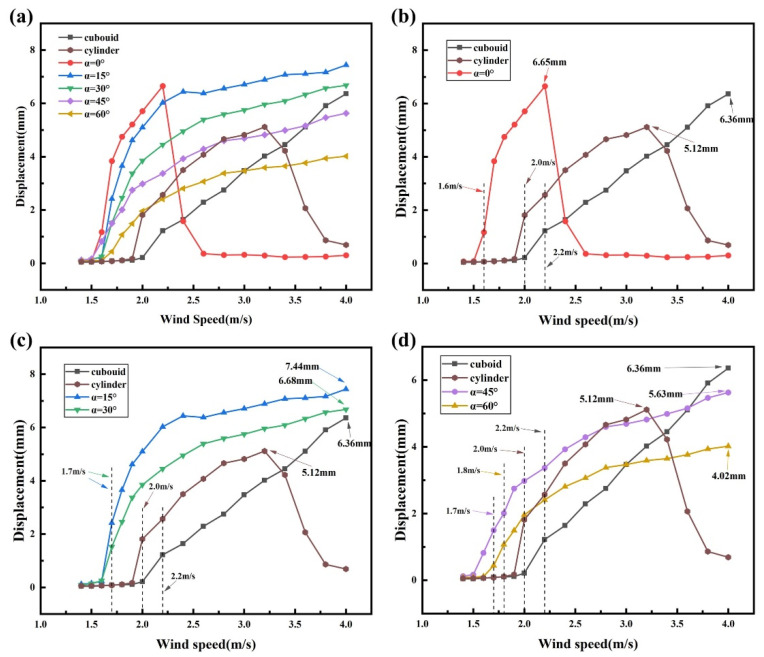
Comparison of transverse vibration amplitudes for BDPEHs with different spreading angles and conventional bluff bodies: (**a**) all BDPEHs versus GPEH and VIVPEH; (**b**) α = 0° versus GPEH and VIVPEH; (**c**) α = 15° and 30° versus GPEH and VIVPEH; (**d**) α = 45° and 60° versus GPEH and VIVPEH.

**Figure 5:**
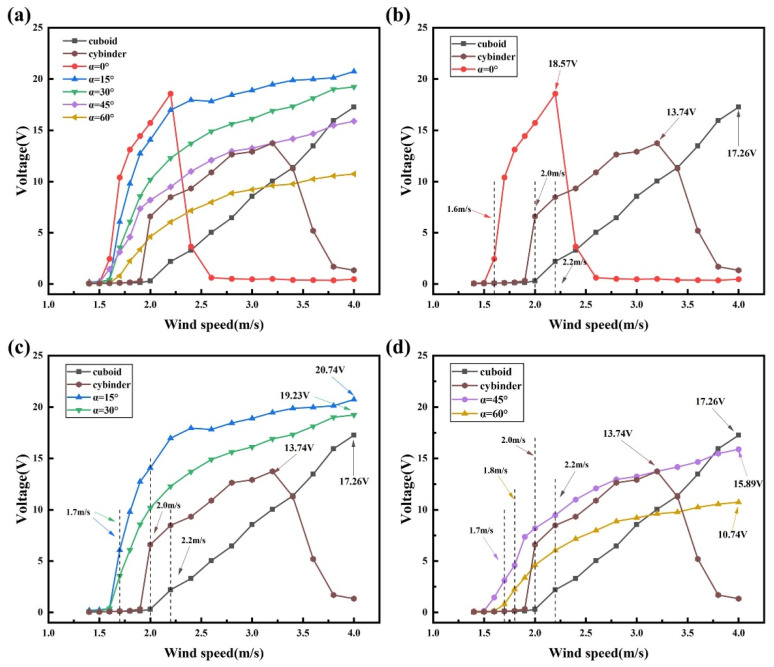
Comparison of RMS voltage outputs for BDPEHs with different spreading angles and conventional bluff bodies: (**a**) all BDPEHs versus GPEH and VIVPEH; (**b**) α = 0° versus GPEH and VIVPEH; (**c**) α = 15° and 30° versus GPEH and VIVPEH; (**d**) α = 45° and 60° versus GPEH and VIVPEH.

**Figure 6:**
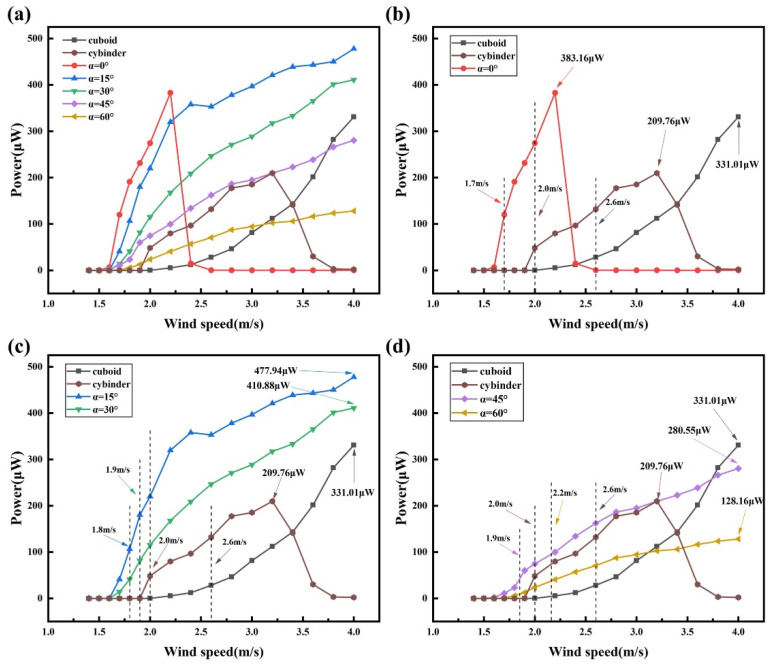
Variation in output power with wind speed for different bluff-body configurations: (**a**) all BDPEHs versus GPEH and VIVPEH; (**b**) α = 0° versus GPEH and VIVPEH; (**c**) α = 15° and 30° versus GPEH and VIVPEH; (**d**) α = 45° and 60° versus GPEH and VIVPEH.

To ensure a fair comparison of electrical output under identical circuit conditions, the external load resistance is fixed at 0.9 MΩ in all experiments. The voltage response, output power, and structural displacement are measured and statistically analyzed, with the results presented in Figure 4, Figure 5 and Figure 6. The root-mean-square voltage (V_rms_) and the output power (P) are adopted as the primary performance metrics, where V_rms_ is calculated from the measured voltage time histories, and the output power is estimated by P = $V_{\mathrm{rms}}^{2}$/R, with R denoting the external load resistance. Considering the target application of the BDPEH under low wind speed conditions, the experimental wind speed range is set to U = 1.4–4.0 m/s.

As the wind speed increases to a critical value, each configuration gradually transitions from a stationary state to transverse vibration. The experimental results show that the cut-in wind speed is approximately 1.6 m/s for α = 0°. For α = 15°, 30°, and 45°, the cut-in wind speed is around 1.7 m/s, whereas it increases to about 1.8 m/s when α reaches 60°. These variations in cut-in wind speed reflect the influence of bluff-body geometry on aerodynamic response characteristics. For comparison, the cut-in wind speed of the VIVPEH is measured to be 2.0 m/s, while the critical galloping wind speed of the GPEH is approximately 2.2 m/s.

From the results shown in Figure 4, Figure 5 and Figure 6, it can be observed that the BDPEH with α = 0° exhibits pronounced VIV characteristics within the wind speed range of 1.6–2.4 m/s. At U = 2.2 m/s, the RMS voltage and output power reach their maximum values within this range, namely 18.57 V and 383.16 μW, respectively. As the wind speed further increases to 2.6 m/s, both the vibration amplitude and electrical output decrease rapidly, indicating that this configuration has departed from the VIV lock-in region. For comparison, the lock-in wind speed range of the VIVPEH is approximately 2.0–3.6 m/s, with maximum RMS voltage and output power of 13.74 V and 209.76 μW, respectively. Although the BDPEH with α = 0° achieves relatively high electrical output at low wind speeds, its effective response range is comparatively concentrated and narrow.

When the wing-opening angle increases to α = 15°, 30°, 45°, and 60°, the vibration displacement, RMS voltage, and output power of the BDPEH exhibit a continuously increasing trend with wind speed and reach their respective maxima near U = 4.0 m/s, indicating response characteristics dominated by galloping. Within the wind speed range of 1.4–4.0 m/s, the maximum RMS voltage and output power of the GPEH are measured to be 17.26 V and 331 μW, respectively. In the wind speed interval of 1.7–4.0 m/s, the BDPEHs with α = 15° and 30° exhibit significantly higher vibration amplitudes and electrical output than both the GPEH and the VIVPEH. For example, at U = 3.0 m/s, the BDPEH with α = 15° delivers an RMS voltage of 18.90 V and an output power of 396.90 μW, while the BDPEH with α = 30° produces 16.12 V and 288.72 μW. Both values are substantially higher than those of the GPEH (8.56 V, 81.41 μW) and the VIVPEH (12.92 V, 185.47 μW).

Within the investigated range of wing-opening angles, the BDPEH with α = 15° exhibits a well-balanced performance in terms of output amplitude and effective operating wind speed range. Its maximum RMS voltage and output power reach 20.74 V and 477.94 μW, representing increases of 20.16% and 44.39% compared with those of the GPEH, and 50.95% and 127.84% compared with those of the VIVPEH, respectively. In terms of onset characteristics, the cut-in wind speed of the α = 15° configuration is reduced by approximately 22.73% and 15.00% relative to the GPEH and VIVPEH, respectively, highlighting its advantage in low-wind-speed operation.

The experimental results further indicate that increasing the wing-opening angle α does not lead to a monotonic improvement in energy harvesting performance. Compared with the GPEH and the VIVPEH, the dragonfly-shaped bluff bodies with α = 45° and 60° exhibit reduced cut-in wind speeds; however, their performance enhancement remains limited over most of the investigated wind speed range. Specifically, the BDPEH with α = 45° exhibits higher output than the VIVPEH only within the wind speed intervals of 1.7–2.8 m/s and 3.4–4.0 m/s, whereas the advantage of the α = 60° configuration mainly appears in the ranges of 1.8–2.0 m/s and 3.6–4.0 m/s, which are largely outside the lock-in region of the VIVPEH. When compared with the GPEH, the α = 45° configuration shows slightly higher output in the wind speed range of 1.7–3.6 m/s, while the advantageous range of the α = 60° configuration further narrows to 1.8–3.0 m/s. Overall, under identical wind speed conditions, the output levels of these two configurations remain lower than those achieved by the α = 15° and 30° cases.

From a structural perspective, this discrepancy may be attributed to changes in the bluff-body cross-sectional proportion and windward projected characteristics as α increases. At larger wing-opening angles, the windward projected area is reduced, and the location of the aerodynamic force application as well as the effective lift distribution are altered, which limits the intensity of aerodynamic excitation over certain wind speed ranges and consequently affects the overall energy conversion efficiency.

A further comparison of the response processes reveals that, in the low wind speed regime, both the VIVPEH and the BDPEH with α = 0° exhibit vibration displacement, RMS voltage, and output power that first increase and then decrease with increasing wind speed. This behavior is consistent with the lock-in characteristics of vortex-induced vibration. As the wind speed continues to increase, the lock-in condition is gradually lost, the vortex-shedding frequency deviates from the natural frequency, and the system response weakens accordingly, exhibiting typical VIV-dominated behavior. In contrast, the vibration response of the GPEH increases in an approximately linear manner with wind speed, without a noticeable amplitude drop. This behavior is more consistent with galloping-dominated responses, where the aerodynamic excitation mainly originates from asymmetric lift forces and exhibits weaker dependence on the structural natural frequency, allowing sustained growth of vibration amplitude over a broader wind speed range.

For the BDPEHs with α = 15°, 30°, 45°, and 60°, an S-shaped variation in vibration response is also observed within the wind speed range of 1.6–2.6 m/s, indicating that the VIV mechanism remains dominant at this stage. When the wind speed exceeds approximately 2.6 m/s, the vibration amplitude no longer decays but instead continues to increase in a quasi-linear manner, revealing a transition from VIV to galloping. This transition is not abrupt but occurs smoothly over a finite wind speed range, enabling the harvester to maintain stable energy output across a broader operating window.

Within the investigated range of wing-opening angles, the BDPEHs with α = 15° and 30° achieve a favorable balance between low-wind-speed onset capability and output stability at moderate wind speeds. By contrast, when α increases to 45° and 60°, although galloping responses can still be triggered at moderate wind speeds, the lift excitation capability does not continue to improve due to changes in windward projected area and increased wake instability. As a result, the overall output performance does not further increase with increasing α.

To compare the dynamic response characteristics of different bluff bodies over the low-to-moderate wind speed range, the BDPEHs with wing-opening angles of α = 0°, 15°, and 30° are selected for experimental comparison with the GPEH and the VIVPEH. The corresponding time–history responses of transverse displacement and RMS voltage are provided in the Supporting Information (Figures S1 and S2), while the main response features are summarized here. At a wind speed of U = 1.9 m/s, neither the GPEH nor the VIVPEH has reached the onset condition, and both exhibit very low voltage output and displacement amplitudes. In contrast, all three BDPEH configurations already display distinct vibration responses, indicating stronger sensitivity to weak aerodynamic excitation. Among them, the α = 15° configuration shows the most pronounced early-stage response. When the wind speed increases to U = 2.2 m/s, the α = 0° configuration reaches its peak response within the VIV lock-in region and exhibits the highest output among the selected configurations. However, as the wind speed further increases to U = 2.6 m/s, the response of the α = 0° configuration rapidly decays, whereas the α = 15° and 30° configurations still maintain comparatively high displacement amplitudes and voltage output. At higher wind speeds, the α = 15° and 30° configurations continue to exhibit relatively stable growth trends, reflecting better response continuity and a wider effective operating range under moderate wind speed conditions.

**Figure 7:**
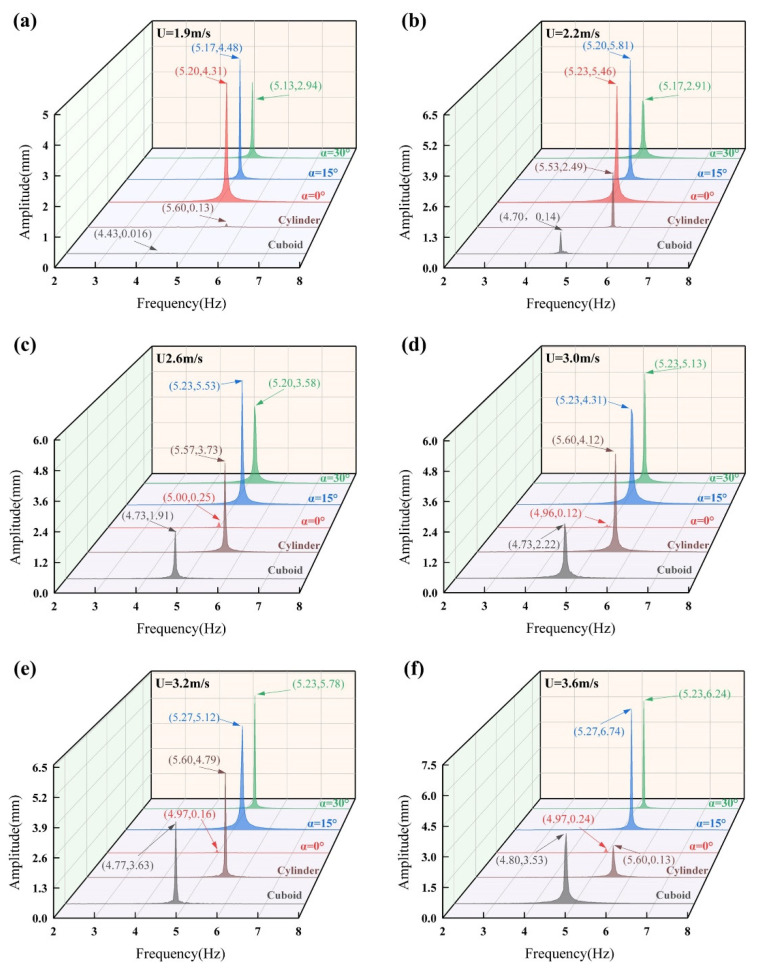
Fast Fourier transform (FFT) spectra of transverse displacement for different bluff bodies at various wind speeds: (**a**) U = 1.9 m/s; (**b**) U = 2.2 m/s; (**c**) U = 2.6 m/s; (**d**) U = 3.0 m/s; (**e**) U = 3.2 m/s; and (**f**) U = 3.6 m/s. The compared cases include BDPEHs with α = 0°, 15°, and 30°, GPEH, and VIVPEH.

To reveal the frequency–domain response characteristics of different bluff-body configurations, fast Fourier transform (FFT) analyses are performed on the vibration displacement signals of the BDPEHs with α = 0°, 15°, and 30°, as well as the GPEH and the VIVPEH, under different wind speed conditions. The corresponding results are presented in Figure 7.

At wind speeds of U = 1.9 m/s and 2.2 m/s, the BDPEHs exhibit clear and well-concentrated dominant frequency components. The dominant frequencies are mainly distributed around 5.2 Hz with relatively large spectral amplitudes, indicating that the system has entered a vortex-induced vibration (VIV)-dominated state. Among the BDPEHs with α = 0°, 15°, and 30°, the dominant frequency locations are close to each other and the spectral peaks are highly concentrated. In contrast, the spectral amplitudes of the GPEH and the VIVPEH remain relatively small, and no pronounced dominant frequency response is observed at these wind speeds.

It is worth noting that, over the wind speed range of U = 1.9–3.6 m/s, the dominant vibration frequencies of the α = 15° and α = 30° configurations remain concentrated within approximately 5.2–5.3 Hz, with only minor variation as wind speed increases. This suggests that the vibration responses of these two configurations remain closely associated with the first natural frequency of the structure over a relatively wide operating range. By contrast, the dominant frequency of the α = 0° configuration shifts from about 5.2 Hz to approximately 5.0 Hz at U = 2.6 m/s and is accompanied by a pronounced reduction in spectral amplitude, quantitatively indicating the gradual loss of lock-in and the weakening of aerodynamic excitation after the VIV-dominated stage.

Within the wind speed interval of U = 3.0–3.6 m/s, the spectral peak amplitude of the α = 0° configuration further decays, and the overall spectral energy is significantly reduced. In contrast, the dominant frequencies of the α = 15° and 30° configurations remain nearly unchanged with increasing wind speed, while their spectral amplitudes are consistently maintained at relatively high levels. Meanwhile, the dominant frequency of the GPEH is mainly concentrated in the range of approximately 4.7–5.0 Hz, although the corresponding amplitude growth is limited. The dominant frequency of the VIVPEH is located at around 5.6 Hz; despite its stable frequency position, the overall spectral amplitude remains comparatively low throughout the investigated wind speed range.

Although FFT analysis is effective in identifying the dominant frequency components and their evolution under different wind speed conditions, frequency–domain information alone is still insufficient to fully characterize the stability of the vibration process. Therefore, the displacement phase portraits under representative wind speed conditions are provided in the Supporting Information (Figures S3 and S4), and their main characteristics are discussed here. For the α = 15° configuration, the phase trajectories remain relatively regular and closed over a relatively wide wind speed range, indicating a stable periodic vibration state and good response continuity. The α = 30° configuration also exhibits closed phase loops, but the enclosed areas are generally smaller and the trajectories become slightly more scattered under some wind speed conditions. In comparison, the α = 0° configuration shows a much stronger dependence on wind speed. At lower wind speeds, its phase trajectories are still relatively clear; however, as the wind speed increases beyond the VIV lock-in region, the loop area decreases rapidly and the trajectories gradually become more dispersed. Among the reference configurations, the phase trajectories of the GPEH remain small in area and poorly concentrated over the entire tested wind speed range, whereas the VIVPEH exhibits relatively regular trajectories but with significantly smaller coverage than those of the BDPEHs with α = 15° and 30°. Overall, from the perspective of phase-space trajectory evolution, the α = 15° configuration exhibits the best dynamic stability among the selected cases, while the α = 30° configuration also maintains a relatively stable vibration state over a certain wind speed range.

**Figure 8:**
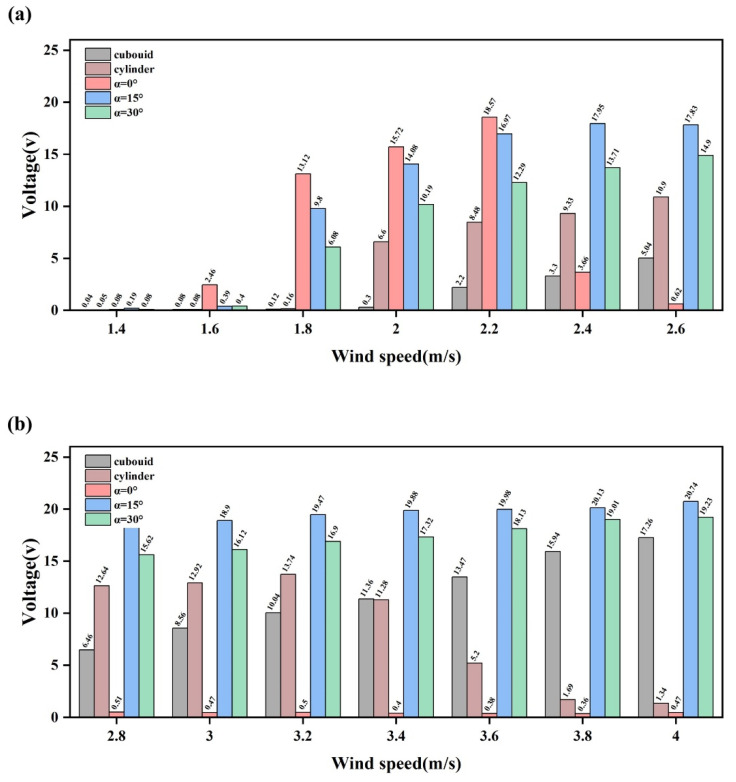
Comparison of RMS voltage responses of different bluff-body configurations under varying wind speeds: (**a**) U = 1.4–2.6 m/s; (**b**) U = 2.8–4.0 m/s. The compared cases include BDPEHs with α = 0°, 15°, and 30°, GPEH, and VIVPEH.

The phase-space analysis indicates that different bluff-body configurations exhibit distinct dynamic characteristics. However, whether such differences in dynamical responses can be effectively translated into electrical output still needs to be evaluated from the perspective of electromechanical response. Figure 8 compares the variations in RMS voltage with wind speed for the BDPEHs with α = 0°, 15°, and 30°, together with the GPEH and the VIVPEH. It can be observed that the different configurations exhibit markedly distinct response evolution characteristics in the low and moderate wind speed regimes. At wind speeds below U = 2.2 m/s, the BDPEH with α = 0° delivers significantly higher RMS voltage levels than the reference bluff bodies. Its peak RMS voltage is approximately 1.07 and 1.35 times those of the cuboid and cylinder configurations, respectively, indicating that this configuration is more capable of establishing effective vibration responses under weak inflow conditions. However, as the wind speed further increases and approaches the upper limit of the vortex-induced vibration lock-in region, namely at U = 2.4 m/s, a pronounced decline in voltage output is observed for the α = 0° configuration, suggesting that its effective operating range is relatively narrow. By contrast, the α = 15° configuration maintains a relatively smooth and continuous increase in RMS voltage throughout the entire tested wind speed range. Under low wind speed conditions, its cut-in wind speed is lower than that of the GPEH by approximately 22.73%. In the moderate wind speed regime, its RMS voltage remains consistently high, with maximum values approximately 20.16% and 50.94% higher than those of the cuboid and cylinder configurations, respectively. These results indicate that the α = 15° configuration is capable of sustaining stable electrical output over a wide range of wind speed conditions.

## 4. CFD Simulations and Flow Mechanism Analysis

The experimental results demonstrate that the BDPEHs with wing-opening angles of α = 15° and 30° are able to maintain stable vibration responses and energy output over a relatively wide wind speed range. In contrast, the response of the α = 0° configuration decays rapidly with increasing wind speed once it departs from the typical VIV lock-in region, while further increasing α to 45° and 60° does not lead to additional performance enhancement. These differences suggest that variations in the wing-opening angle may significantly influence the system response by altering wake structures and aerodynamic excitation mechanisms. To further elucidate the flow-induced origins of the performance differences among different configurations, computational fluid dynamics (CFD) simulations are conducted to analyze the near-field flow characteristics under low wind speed conditions.

The numerical simulations are performed using the ANSYS Fluent 2022 R1, where two-dimensional unsteady calculations are employed to describe the flow around the bluff bodies. Based on the experimentally representative configurations, the dragonfly-shaped bluff bodies with α = 0°, 15°, and 30° are selected as the primary cases, while the cuboid and cylinder bluff bodies are introduced as reference models to examine the influence of geometric differences on flow characteristics and aerodynamic responses. In addition, a classical D-shaped bluff body is included as a geometric reference to assist in identifying the coupling characteristics between vortex-induced vibration and galloping.

The simulations focus on near-field flow behaviors under low wind speed conditions, including shear-layer development, wake vortex shedding, and pressure distribution variations. To achieve a balance between computational efficiency and the ability to resolve unsteady flow features, a two-dimensional computational domain is adopted, and the shear stress transport (SST) k–ω turbulence model is employed to close the Reynolds-averaged governing equations. Although the experimental structure has a finite spanwise height, the present numerical analysis mainly focuses on the dominant two-dimensional near-wake features associated with transverse aerodynamic excitation, including boundary-layer separation, periodic vortex shedding, and pressure fluctuation patterns. Under the investigated Reynolds number condition, a two-dimensional model is effective for capturing the primary vortex-shedding characteristics and for comparing the relative aerodynamic behaviors of different bluff-body cross sections. Therefore, the present approach provides a reasonable balance between physical interpretability and computational efficiency. This turbulence model has demonstrated good capability in predicting attached–separated flows and wake dynamics and has been adopted in studies related to flow-induced vibration [57].

During the preprocessing stage of the numerical simulations, a two-dimensional computational domain centered on the dragonfly-shaped bluff body is established. The domain is arranged as a rectangular region with a length of 2500 mm and a width of 800 mm, corresponding to 50 and 16 times the characteristic dimension (50 mm), respectively, in order to minimize the influence of boundary conditions on wake development. A locally refined mesh region with dimensions of 1200 mm × 400 mm is introduced around the bluff body and in the downstream wake region to better resolve shear-layer evolution and wake vortex structures, thereby improving capture accuracy. The overall mesh mainly consists of structured quadrilateral elements. Boundary-layer inflation grids are applied along the bluff-body surface and in the wake region, ensuring a balance between near-wall flow resolution and computational efficiency.

**Figure 9:**
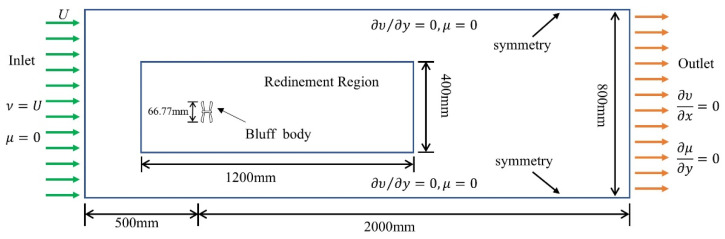
Computational domain and boundary conditions adopted in the CFD simulations.

For boundary conditions, a velocity inlet is specified at the upstream boundary with an incoming wind speed of U = 2.5 m/s, while a pressure outlet is imposed at the downstream boundary. The inlet wind speed in the CFD simulations is set to U = 2.5 m/s as a representative operating condition in the low-to-moderate wind speed regime. At this condition, the corresponding Reynolds number remains within the subcritical range, while the aerodynamic response differences among the bluff-body configurations have already become evident. In particular, this condition is close to the transition region where the α = 0° configuration begins to depart from the VIV lock-in state, whereas the α = 15° and 30° configurations still maintain comparatively stable responses. Therefore, this simulation condition is adopted to provide a representative basis for flow-field comparison and mechanism interpretation. Symmetry conditions are applied to both the upper and lower boundaries of the computational domain. The bluff body is positioned 500 mm downstream from the inlet and centered in the transverse direction of the computational domain. Under this condition, the corresponding Reynolds number is Re = 8557, which lies within the subcritical flow regime. In this regime, relatively stable periodic vortex shedding can develop in the wake, inducing alternating negative pressure distributions on the upper and lower surfaces of the bluff body and resulting in pronounced lift fluctuations. The detailed configuration of the computational domain, refined mesh region, and boundary conditions is illustrated in Figure 9.

To assess the sensitivity of the numerical results to mesh resolution and to achieve a balance between accuracy and computational cost, three meshes with different resolutions—coarse, medium, and fine—are examined, containing 48,226, 71,410, and 149,098 cells, respectively. The root-mean-square value of the lift coefficient (C_L_) and the time-averaged value of the drag coefficient (C_D_) are adopted as evaluation metrics to compare the aerodynamic responses under different mesh conditions, and the results are summarized in Table 1. The results indicate that the main aerodynamic characteristics gradually converge with mesh refinement. The differences between the medium and fine meshes are already negligible; therefore, the medium mesh is adopted for all subsequent simulations.

**Figure 10:**
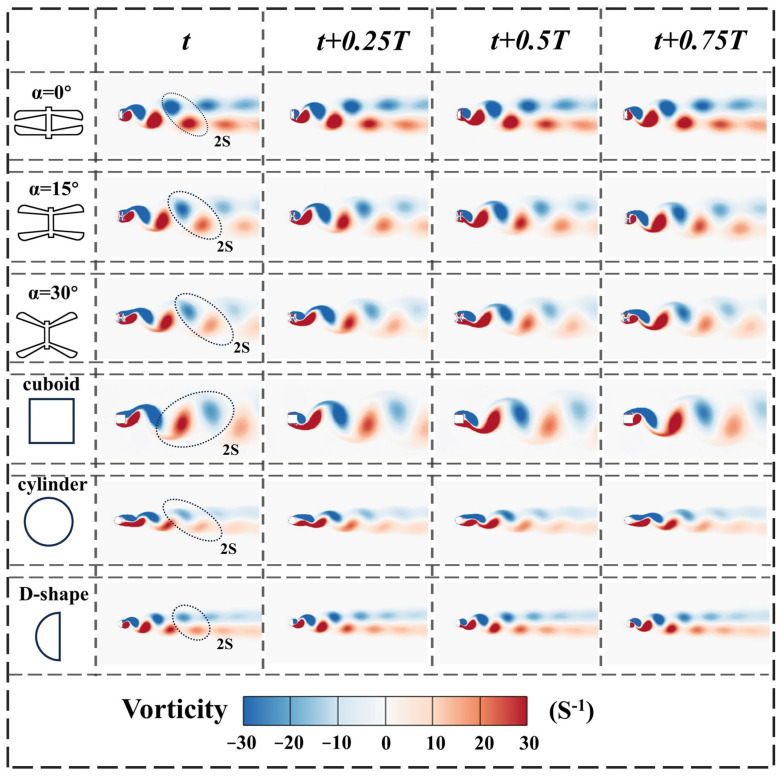
Instantaneous near-wake vortex structures obtained from CFD simulations for BDPEHs with α = 0°, 15°, and 30°, GPEH, VIVPEH, and the D-shaped bluff body, shown at four representative instants within one complete vortex-shedding cycle: t, t + 0.25T, t + 0.5T, and t + 0.75T.

**Figure 11:**
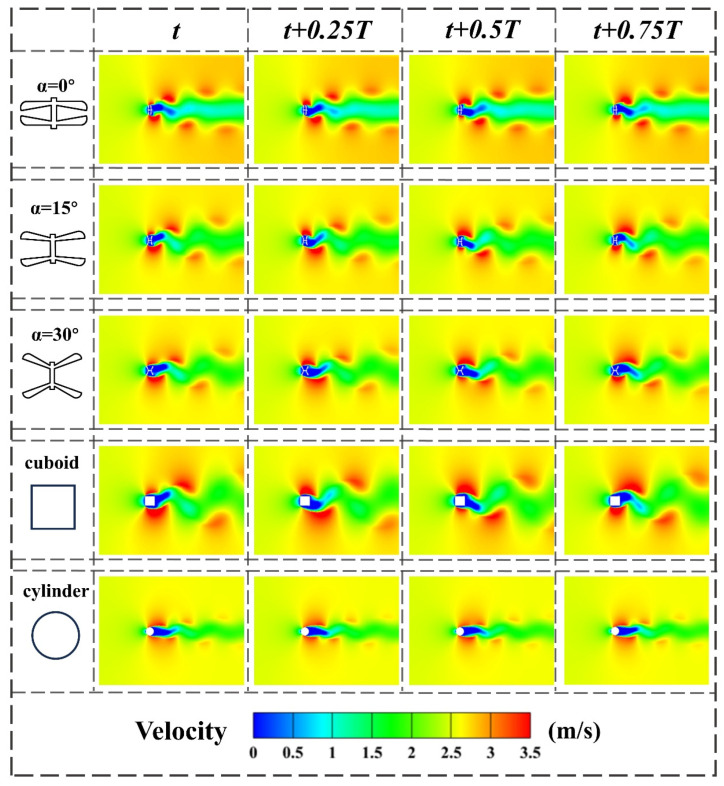
Instantaneous velocity contours obtained from CFD simulations for BDPEHs with α = 0°, 15°, and 30°, GPEH, and VIVPEH, shown at four representative instants within one complete vortex-shedding cycle: t, t + 0.25T, t + 0.5T, and t + 0.75T.

**Figure 12:**
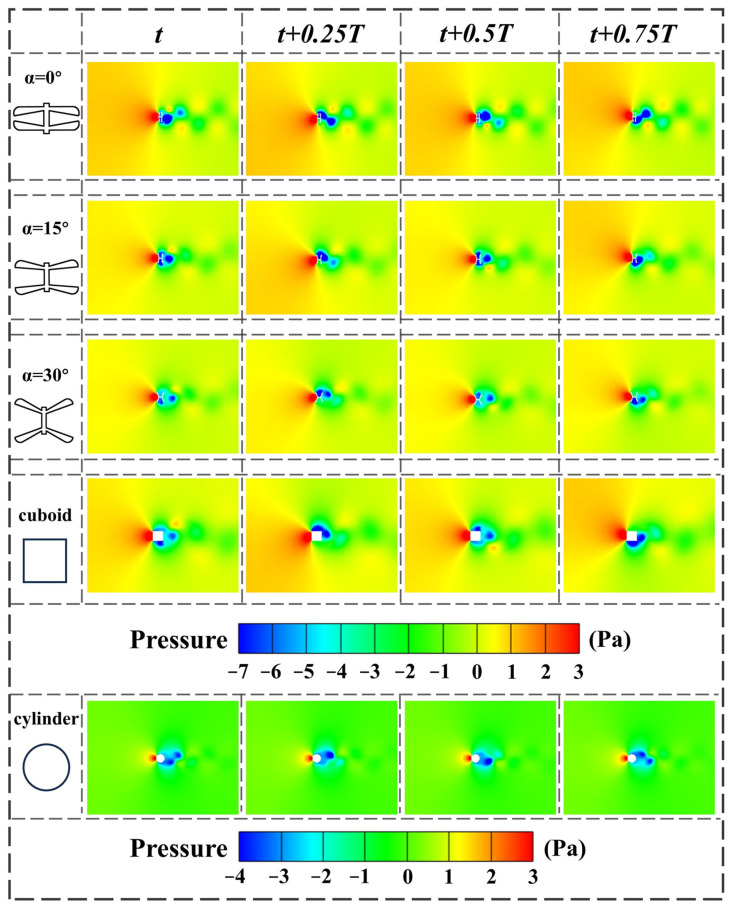
Instantaneous pressure distribution contours obtained from CFD simulations for BDPEHs with α = 0°, 15°, and 30°, GPEH, and VIVPEH, shown at four representative instants within one complete vortex-shedding cycle: t, t + 0.25T, t + 0.5T, and t + 0.75T.

After obtaining the CFD results for the dragonfly-shaped bluff body, the cuboid bluff body, the cylinder bluff body, and the D-shaped bluff body, flow-field visualization is conducted. Post-processing is performed using Tecplot 360 EX 2023 R1, and the flow characteristics over a complete vortex-shedding cycle are analyzed in terms of vorticity, velocity vectors, and pressure distribution, as shown in Figure 10, Figure 11 and Figure 12.

The vorticity contours in Figure 10 reveal pronounced differences among the bluff bodies in terms of vortex formation locations, shedding trajectories, and wake evolution patterns. Owing to its sharp windward edges, the cuboid bluff body experiences early flow separation near the leading edges, generating large-scale vortical structures in the wake, accompanied by rapid vorticity diffusion and relatively weak wake stability. For the cylinder bluff body, the separation point is located further downstream, resulting in a relatively regular Kármán vortex street in the wake, although the overall vorticity intensity remains limited.

Under the present operating condition, the D-shaped bluff body exhibits vortex-shedding characteristics intermediate between those of the cuboid and cylinder configurations. Vortices are mainly generated near the leeward side and shed downstream along relatively concentrated paths. The wake retains a certain degree of periodicity while exhibiting pronounced asymmetry, which is commonly regarded as a typical flow feature associated with coupled vortex-induced vibration and galloping.

By comparison, the dragonfly-shaped bluff bodies with α = 0°, 15°, and 30° show a high degree of similarity to the D-shaped bluff body in terms of vortex-shedding trajectories. Vortices are predominantly generated near the trailing edges of the wing structures and shed alternately along directions guided by the wing plates. In particular, under the α = 15° and 30° conditions, the alternating vortex rows maintain well-defined structures over a relatively long downstream distance, indicating improved wake organization. These observations suggest that the wake flow of the dragonfly-shaped bluff body is not governed by a single VIV or galloping mechanism, but rather corresponds to a transitional state resulting from the synergistic interaction of both mechanisms. This feature is consistent with the smooth transition of vibration responses observed in the experiments.

Regarding the vortex-shedding frequency, the shedding period of the cuboid bluff body is approximately 10 time steps, while that of the cylinder is about 5 time steps, corresponding to typical galloping-dominated and VIV-dominated behaviors, respectively. For the dragonfly-shaped bluff bodies, the shedding periods of the α = 0° and 15° configurations are approximately 6 time steps, whereas the period increases to about 8 time steps for α = 30°. The shedding frequency of the D-shaped bluff body also falls within this range, indicating that this frequency interval can be regarded as a transitional region linking typical VIV- and galloping-dominated behaviors.

Further comparison of the time histories of the lift coefficient reveals that, under identical inflow conditions, the dragonfly-shaped bluff bodies generate lift fluctuations with overall higher amplitudes and clearer periodicity than the D-shaped bluff body. This indicates that the dragonfly-shaped configuration is capable of supplying stronger and more organized transverse aerodynamic forces to the structure, which is beneficial for reducing the onset threshold at low wind speeds and maintaining large-amplitude vibration responses in the moderate wind speed regime. It should be noted that, once the α = 0° configuration departs from the lock-in region, both the amplitude of lift fluctuations and the stability of their periodicity decrease markedly, accompanied by a gradual loss of wake organization. This behavior is consistent with the rapid decay of energy output observed in the experiments.

In addition, under the present operating condition, both the dragonfly-shaped bluff body and the cuboid configuration exhibit a typical 2S vortex-shedding mode, in which vortices are alternately shed from the upper and lower surfaces, generating periodic pressure differences across the structure. When α = 15°, the dragonfly-shaped bluff body achieves a more balanced state between geometric transition and windward projected characteristics. This allows the incoming flow to slide more smoothly along the surface, leading to relatively stable boundary-layer separation locations and facilitating the formation of balanced shear layers on the upper and lower surfaces. As a result, a continuous and regular alternating vortex-shedding process can be sustained.

The velocity contours shown in Figure 11 reveal pronounced differences in wake-flow patterns among the various bluff-body geometries. Behind the dragonfly-shaped bluff body, a continuous oscillatory velocity channel is formed, in which the high-speed region undergoes periodic lateral oscillation along the centerline, while the downstream velocity recovery remains relatively smooth. This behavior indicates that the evolution of the shear layers and the vortex-shedding process exhibit good stability. In contrast, the wake of the cuboid bluff body shows a velocity distribution biased toward one side, with large disturbance amplitudes and weak spatial coherence. The cylinder bluff body forms a more classical Kármán vortex street, characterized by a relatively narrow velocity channel with good symmetry but limited oscillation amplitude.

The pressure contours further highlight the differences in aerodynamic loading among the configurations. During a single shedding cycle, the dragonfly-shaped bluff body maintains a clear pressure difference between the upper and lower surfaces, with high pressure concentrated near the leading edge and alternating low-pressure regions periodically appearing near the trailing edge. Low-pressure cores in the wake subsequently develop downstream in sequence, corresponding to a typical 2S vortex-shedding mode. For the cuboid bluff body, the pressure distribution exhibits strong asymmetry, with pronounced temporal fluctuations in pressure difference. In contrast, the pressure field around the cylinder bluff body is relatively symmetric, but the intensity of the low-pressure regions is limited, resulting in a comparatively weak transverse driving force.

By jointly examining the velocity and pressure fields, it can be concluded that the dragonfly-shaped bluff body with α = 15° achieves a well-coordinated spatiotemporal distribution between wake structures and pressure fluctuations. This provides a flow-field-based explanation for its low cut-in wind speed and stable energy output observed in the experiments.

## 5. Conclusions

This study addresses the efficiency limitations of flow-induced vibration-based wind energy harvesting under low wind speed conditions by proposing and investigating a bio-inspired dragonfly-wing-based bluff-body piezoelectric energy harvester. Through a combined approach of wind tunnel experiments and CFD simulations, the aerodynamic responses and energy harvesting performances of dragonfly-shaped bluff bodies with different wing-opening angles were systematically examined over a low-to-moderate wind speed range. Based on the experimental observations and flow-field analyses, the main conclusions can be summarized as follows:

1. Within the tested wind speed range, all dragonfly-shaped bluff bodies exhibited superior low-wind-speed initiation characteristics compared with conventional configurations. Stable vibrations were achieved at significantly lower wind speeds than those required for the cylinder-based harvester (VIVPEH, 2.0 m/s) and the cuboid-based harvester (GPEH, 2.2 m/s). Specifically, the cut-in wind speed was reduced to 1.6 m/s for α = 0°, to 1.7 m/s for α = 15°, 30°, and 45°, and to 1.8 m/s for α = 60°, indicating more favorable aerodynamic excitation conditions induced by the dragonfly-inspired geometry.
2. Among all dragonfly-shaped configurations, the wing-opening angle α = 15° delivered the best overall energy harvesting performance. The maximum RMS voltage and output power reached 20.74 V and 477.94 μW, corresponding to increases of 20.16% and 44.39% relative to the GPEH, and 50.95% and 127.84% relative to the VIVPEH, respectively. At a wind speed of U = 3.0 m/s, the RMS voltage of the α = 15° configuration exceeded those of the cuboid and cylinder configurations by approximately 120.79% and 46.28%, while the corresponding output power increased by about 387.53% and 113.99%. In contrast, the α = 0° configuration exhibited relatively high output only within the narrow VIV lock-in range of 1.6–2.2 m/s, with peak RMS voltage and power increases of 7.59% and 15.76% compared with the GPEH, and 35.15% and 82.67% compared with the VIVPEH, indicating strong dependence on the VIV lock-in mechanism. For configurations with α ≥ 45°, the overall performance improvement was limited.
3. Time–domain and frequency–domain analyses revealed that the dragonfly-shaped bluff bodies with α = 15° and 30° could achieve a smooth transition from vortex-induced vibration to galloping as wind speed increased. This transition effectively avoids the rapid response decay commonly observed in single-mode VIV systems after lock-out, enabling sustained large-amplitude vibrations and stable energy output over a wider operational wind speed range.
4. CFD simulations provided mechanistic insight into the experimentally observed performance differences. The dragonfly-inspired wing-opening geometry effectively regulates shear-layer development and vortex-shedding paths in the near wake, resulting in vortex-shedding frequencies distributed between the characteristic ranges of typical VIV and galloping. This flow behavior facilitates the synergistic interaction of the two aerodynamic excitation mechanisms. Among the investigated configurations, α = 15° achieves a favorable balance between wake stability and lift fluctuation intensity, which explains its superior overall performance.

In summary, by tuning the wing-opening angle, the bio-inspired dragonfly-shaped bluff body enables effective control of aerodynamic excitation modes, simultaneously enhancing cut-in capability and output stability under low wind speed conditions. The present study provides valuable guidance for structural design and parameter optimization of distributed wind energy harvesters operating in low-wind environments, and offers new insights into the application of bio-inspired aerodynamic structures for flow-induced vibration energy harvesting. From an engineering application perspective, the proposed dragonfly-wing-inspired harvester shows promise for powering distributed low-power devices, such as wireless sensor nodes, in outdoor low-wind environments. Owing to its improved cut-in capability and stable output over a relatively wide wind speed range, the harvester is expected to provide a more reliable long-term energy supply under practical ambient wind conditions.

## Figures and Tables

**Table 1:** Mesh independence verification.

Mesh Sizes	Number of Cells	*C*_L,rms_	*C*_D,mean_
Coarse mesh	48,226	0.6709	0.9291
Medium mesh	71,410	0.6768 (+0.8%)	0.9214 (−0.83%)
Fine mesh	149,098	0.6691 (−1.13%)	0.9105 (−1.18%)

## Data Availability

The data that support the findings of this study are available upon reasonable request from the authors.

## Supplementary Materials

The following supporting information can be downloaded at: https://www.mdpi.com/article/10.3390/mi17030380/s1, Figure S1: Time-history responses of transverse displacement for the BDPEHs with α = 0°, 15°, and 30°, together with the GPEH and the VIVPEH, at U = 1.9, 2.2, 2.6, and 3.0 m/s; Figure S2: Time-history responses of RMS voltage for the BDPEHs with α = 0°, 15°, and 30°, together with the GPEH and the VIVPEH, at U = 1.9, 2.2, 2.6, and 3.0 m/s; Figure S3: Phase portraits of transverse displacement for the BDPEHs with α = 0°, 15°, and 30°, together with the GPEH and the VIVPEH, at representative wind speeds; Figure S4: Phase portraits of transverse displacement for the BDPEHs with α = 0°, 15°, and 30°, together with the GPEH and the VIVPEH, under additional representative wind speed conditions.
