# Supplementary material for: Dragonfly-Wing-Inspired Bluff-Body Piezoelectric Harvester for Efficient Low-Wind-Speed Energy Harvesting
Source: Micromachines (Basel). 2026 Mar 20;17(3):380. doi: 10.3390/mi17030380 (PMC13029154; doi:10.3390/mi17030380)
Supplement: Supplementary file 1 [file micromachines-17-00380-s001.zip › micromachines-4169862-supplementary.pdf]

Supporting Information

Supporting Information for

"Dragonfly-wing-inspired bluff-body piezoelectric harvester for efficient low-wind-speed energy harvesting"

This document contains supplementary figures referred to in the main manuscript as Figs.S1-S4.

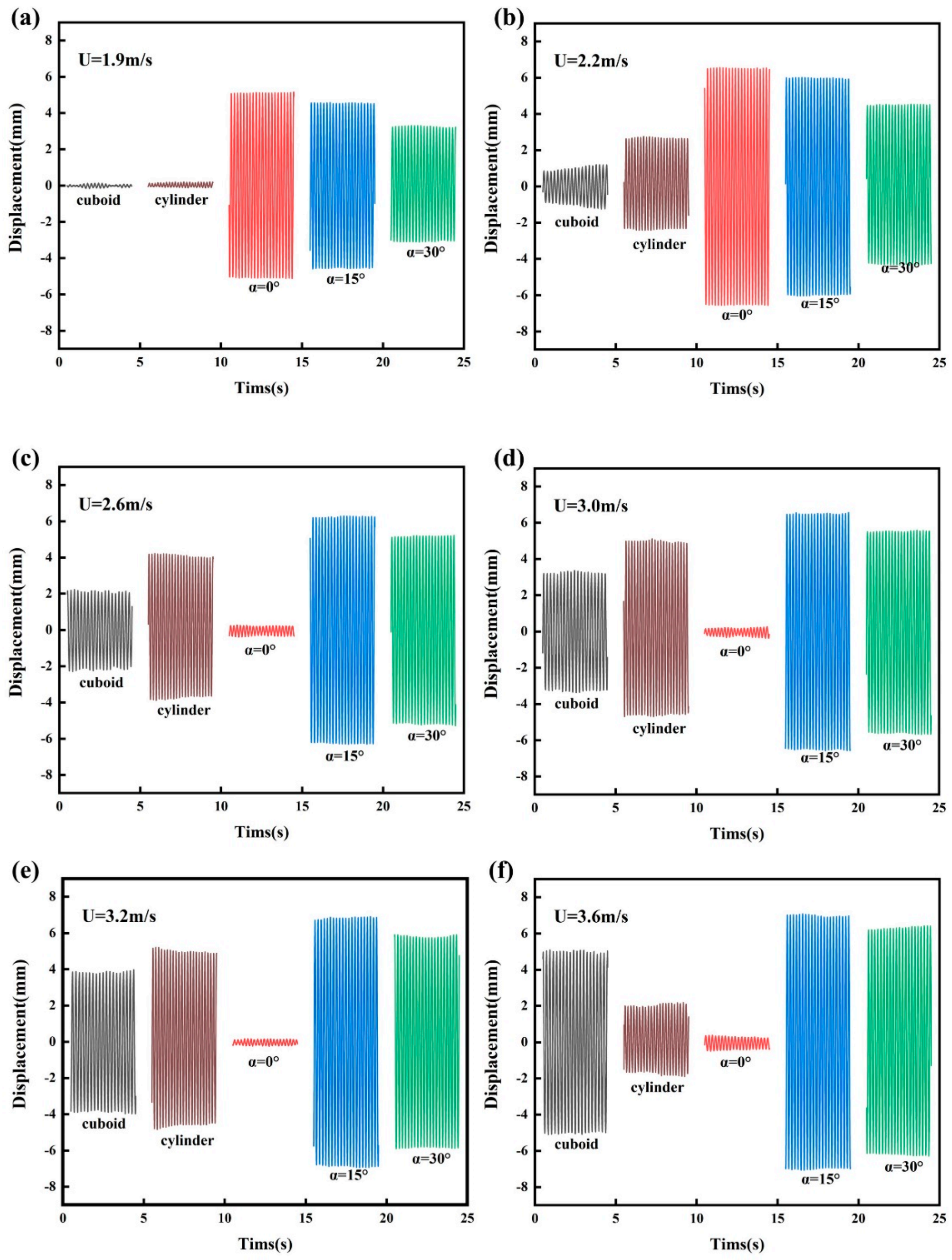

Figure S1. Time-history responses of transverse displacement for the BDPEHs with α = 0°, 15°, and 30°, together with the GPEH and the VIVPEH, at U = 1.9, 2.2, 2.6, and 3.0 m/s.

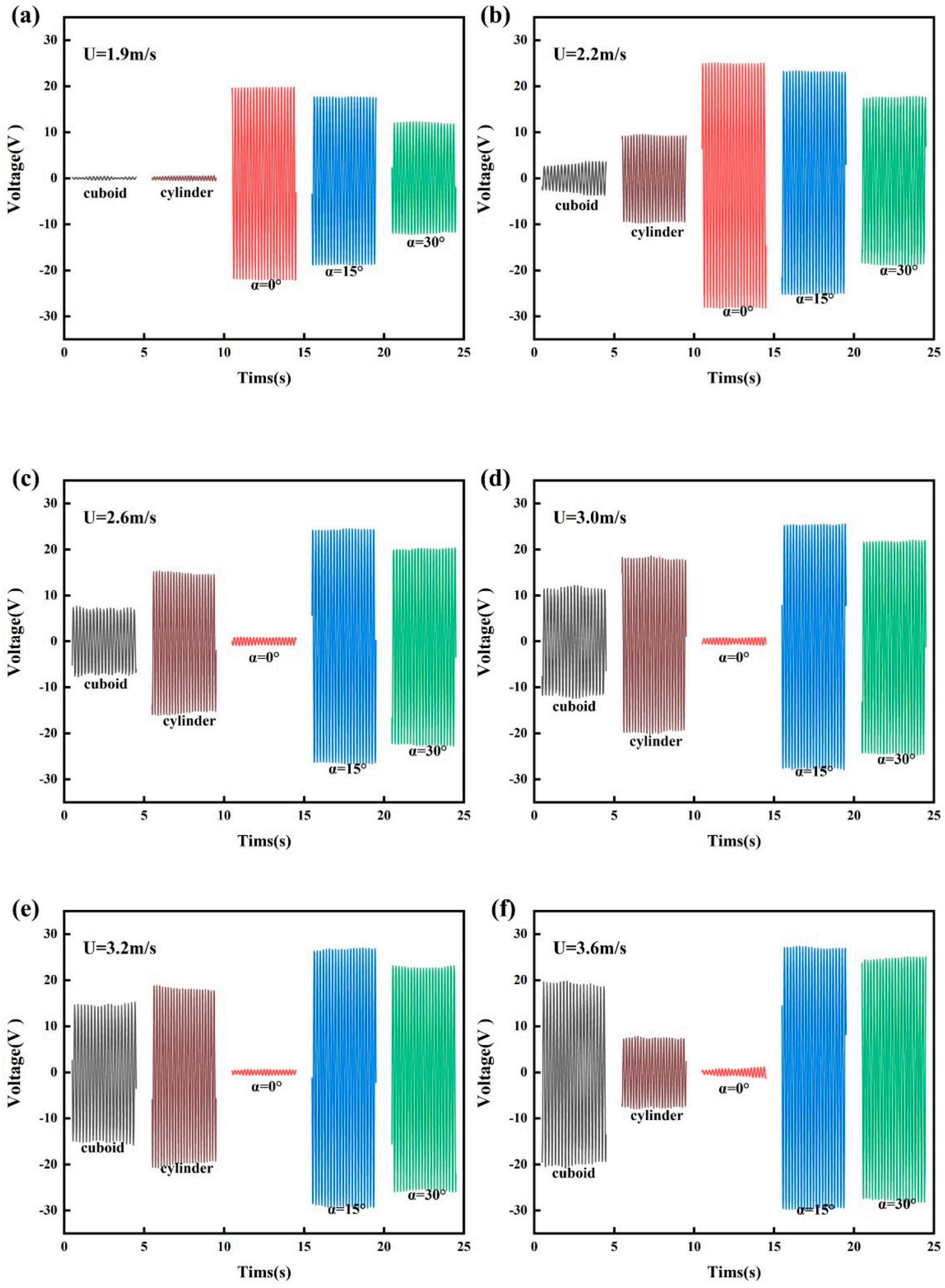

Figure S2. Time-history responses of RMS voltage for the BDPEHs with α = 0°, 15°, and 30°, together with the GPEH and the VIVPEH, at U = 1.9, 2.2, 2.6, and 3.0 m/s.

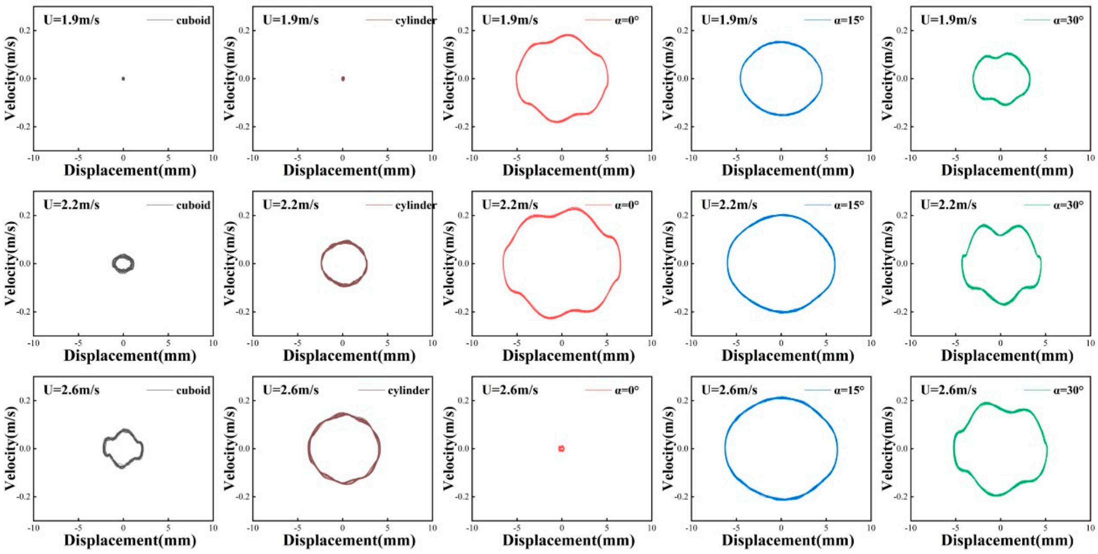

Figure S3. Phase portraits of transverse displacement for the BDPEHs with α = 0°, 15°, and 30°, together with the GPEH and the VIVPEH, at representative wind speeds.

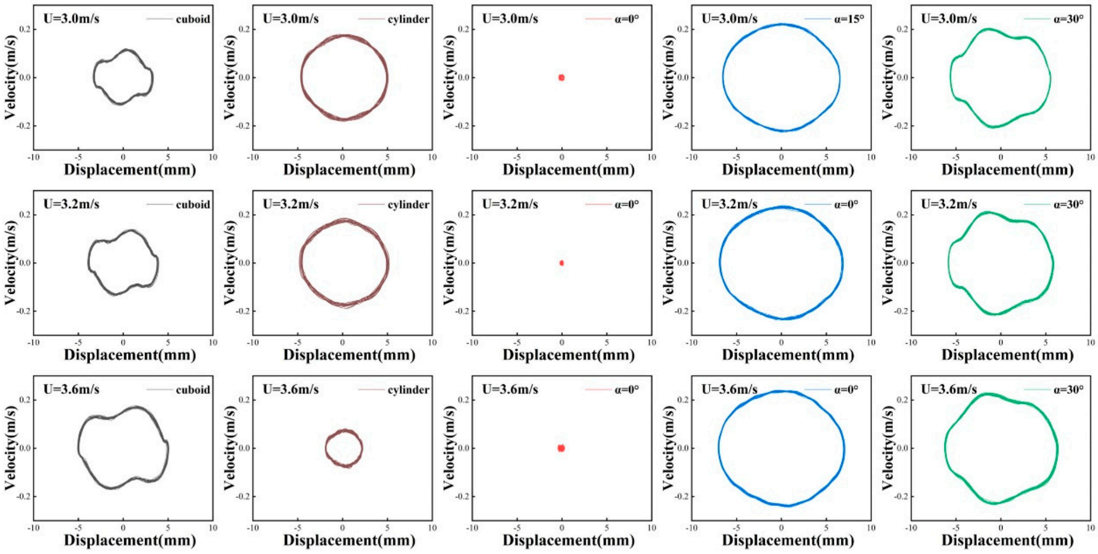

Figure S4. Phase portraits of transverse displacement for the BDPEHs with α = 0°, 15°, and 30°, together with the GPEH and the VIVPEH, under additional representative wind speed conditions.
